# Clinical application of single incision thoracoscopic surgery: early experience of 264 cases

**DOI:** 10.1186/1749-8090-9-44

**Published:** 2014-03-08

**Authors:** In-Hag Song, Sungwon Yum, Wonsuk Choi, Sukki Cho, Kwhanmien Kim, Sanghoon Jheon, Hee Chul Yang

**Affiliations:** 1 Department of Thoracic and Cardiovascular Surgery, Seoul National University Bundang Hospital, Seoul National University College of Medicine, 166 Gumi-ro, Bundang-gu, Seongnam-si, Gyeonggi-do 463–707, Seoul, Korea

**Keywords:** VATS, Single incision thoracoscopic surgery, Uniportal, Pneumothorax, Single port surgery

## Abstract

**Background:**

Single incision thoracoscopic surgery (SITS) is recognized as a difficult procedure and surgeons hesitate to perform this technique. We describe our experience of SITS and determine whether SITS can be a routine approach in minimally invasive surgery.

**Methods:**

From May 2011 to April 2013, a single operator attempted SITS for 264 cases. Their medical records were retrospectively reviewed with regard to age, sex, diagnosis, operation time, hospital stay, need of additional incision, morbidity, and early outcome.

**Results:**

A number of thoracic diseases and procedures were attempted with SITS including primary (n = 172) or secondary (n = 22) spontaneous pneumothorax, biopsy for lung (n = 29), pleura (n = 3), and mediastinal lymph node (n = 3), mediastinal mass excision (n = 11), empyema decortication (n = 11), lobectomy (n = 6), pulmonary metastasectomy (n = 3), pericardial window formation (n = 3), and hematoma evacuation (n = 1). Of these, 237 cases underwent SITS successfully. However, additional incision was needed in 10.2% (n = 27). Reasons for conversions were as follows: extensive pleural adhesion (n = 14), difficulty in endoscopic stapling (n = 11), bleeding (n = 1), and intolerance of one lung ventilation (n = 1). Conversion rate of empyema was 54.5%, which was the most difficult for SITS. In contrast, the conversion rate of PSP was 4.7%, which means PSP was the most applicable for SITS. Postoperative complications included air leak (≥3 days) (n = 1), wound problem (n = 4), delayed pleural effusion (n = 1), and postoperative bleeding (n = 1).

**Conclusions:**

SITS can be a routine approach from simple to more complicated diseases. However, we still have difficulties in cases with extensive pleural adhesion or location of lesion with difficult accessibility for endoscopic stapling.

## Background

Video-assisted thoracic surgery (VATS) has been performed from the late 1980s. Since then, the technique has widened its clinical indications for the past 20 years. Numerous previous studies have already proved that VATS is more advantageous in postoperative recovery, cosmesis and also oncological aspect compared to the conventional thoracotomy [1-4]. Furthermore, as surgical experiences in VATS accumulated and instruments advanced, there were attempts to perform single incision thoracoscopic surgery (SITS) as an alternative for the multiport VATS, and already a few studies have reported on the safety and feasibility of SITS [5-10].

Our previous study has attested, by comparing SITS and the conventional three-port VATS, that SITS can be applied as a routine procedure in primary spontaneous pneumothorax (PSP) patients. This was because SITS proved to decrease paresthesia of chest wall and increase satisfaction rate for the wound scar with high reproducibility [10]. However, previous studies on SITS including our results have reported on the basis of a small case series [5-7,9-14]. Currently, we are at the threshold where the technique needs to collect more evidences to make grounds for wider application of SITS.

In our hospital, we did not limit SITS in only PSP patients, but also actively performed in other thoracic diseases. As a result, we attempted SITS for 264 cases with successful 237 cases, which records as second highest number of cases reported by single operator until now [15]. We reviewed our early experiences with the use of SITS in the treatment of various thoracic diseases and evaluated whether SITS can be a routine approach in minimally invasive thoracic surgery.

## Methods

In 2009, Rocco suggested the indications of SITS as a general thoracic surgical practice [8]. In May 2011, we first applied SITS when performing pulmonary wedge resection in a patient with PSP. Since then, as Rocco had suggested, we continued to widen surgical indications with the accumulation of surgical experiences.

A single operator (HCY) at Seoul National University Bundang Hospital performed 491 thoracic surgical procedures from May 2011 to April 2013 and the medical records were reviewed retrospectively. SITS was prior consideration for all VATS cases during the period. There were 162 surgeries which had nothing to do with VATS. Among the remaining 329 VATS procedures, SITS was attempted in a total of 264 (264/329; 80.2%) surgeries. We performed SITS in patients with primary or secondary pneumothorax, well demarcated mediastinal mass, empyema, diffuse interstitial lung disease that need lung biopsy, indeterminate peripheral pulmonary nodule, parietal pleural nodule, pericardial effusion, mediastinal lymph node enlargement, bronchiectasis, aspergilloma, congenital abnormality of the lung and even with primary lung cancer (Table 1). Although the surgery was performed with VATS, 65 cases which did not implement SITS in the first place were excluded. The exceptions, where SITS could not be applied were as below: pneumothorax patients with previous 3-port VATS wound scar (n = 4), patients with pneumothorax (n = 17) or empyema (n = 12) with large bore chest tube in a position that was not appropriate for SITS, empyema patients with peel of 10 mm or greater in the computed tomography (CT) scan (n = 7), patients with mediastinal tumor mass of greater than 9 cm (n = 2), patient with tumor located between the superior vena cava and the innominate vein posing risks for the procedure (n = 1), thymoma patients with myasthenia gravis (n = 2), patient with diaphragmatic hernia (n = 1), patients with compromised lung function before lung biopsy surgery (n = 9), patient who scheduled VATS lobectomy and other organ cooperative operation while minimizing the operation time (n = 1), those who required finger palpation of the mass when doing a wedge resection for pulmonary nodule (n = 3), two or more locations of lesions that were difficult to access via SITS (n = 4), and when the patients who required VATS lobectomy were predicted to have anthracofibrotic lymph nodes in the preoperative CT and bronchoscopy (n = 2). The collected data were age, sex, diagnosis, operation time, hospital stay, need of additional incision, postoperative complications, and short-term outcome. This study was approved by Institutional Review Board of our institution with waiver of informed consent (SNUBH ID: B-1206-158-113).

**Table 1:** Indication of single incision thoracoscopic surgery

**Type of thoracic disease**		**Operation**	**Additional incision**	**Comment**
Pneumothorax (n = 194)	PSP (n = 172)	Resection of blebs/bullae	n = 8, 4.7%	
	SSP (n = 22)		n = 4, 18.2%	
Mediastinal tumor	Thymic cyst (n = 4)	Mass excision	Neurogenic tumor (n = 1, 9.1%)	Tumor size
Anterior (n = 7)	Thymoma (n = 1)			(R: 1.2-6.7 cm)
Posterior (n = 4)	Pericardial cyst (n = 1)			
	Neurogenic tumor (n = 4)			
	Mature teratoma (n = 1)			
Interstitial lung disease (n = 21)	RUL (n = 3)	Wedge resection	n = 4, 19%	
	RML (n = 1)	1 site (n = 15)	RLL 1	
	RLL (n = 5)	2 site (n = 6)	RUL & RLL 1	
	LUL (n = 2)		RML & RLL 1	
	LLL (n = 4)		LUL & LLL 1	
	RUL & RLL (n = 2)			
	RML & RLL (n = 1)			
	LUL & LLL (n = 3)			
Solitary pulmonary nodule (n = 5)	RML (n = 1)	Wedge resection	n = 2, 40%	
	RLL (n = 2)		RLL 1	
	LUL (n = 2)		LUL 1	
Empyema (n = 11)	Non-TB (n = 10)	Decortication	n = 6, 54.5%	
	TB (n = 1)			
Mediastinal	4R (n = 1)	Lymph node biopsy	n = 2, 66.7%	Reason: bleeding
lymphadenopathy (n = 3)	5 (n = 1)			LN location
	6,7 (n = 1)			
Pleural seeding and/or pulmonary metastases (n = 6)	Lung cancer (n = 3)	Biopsy of parietal pleura and/or pleurodesis Biopsy of lung	0	
	Metastases (n = 3)			
Pericardial effusion (n = 3)	Unknown etiology (n = 1)	Pericardial window formation	0	6^th^ ICS approach, left
	Post CT (n = 1)			
	Postoperative (n = 1)			
Lung cancer (n = 3)	RUL (n = 2)	Lobectomy & mediastinal LN dissection	0	4-5 cm skin incision
	RLL (n = 1)			
Inflammatory lung disease (n = 6)		Lobectomy	0	2.5 cm skin incision
	Bronchiectasis (n = 1)	:LLL		
	Aspergillosis (n = 1)	:RUL		
	Lobar emphysema (n = 1)	:LUL		
	Tuberculosis (n = 3)	Biopsy of lung		
Postoperative bleeding (n = 1)		Hematoma evacuation	0	Due to mechanical pleurodesis

PSP, Primary Spontaneous Pneumothorax; SSP, Secondary Spontaneous Pneumothorax; R, range; TB, Tuberculosis; LN, Lymph node; ICS, Intercostal space; CT, Chemotherapy.

### Surgical technique

The patient was in a lateral decubitus position after intubation with double lumen endotracheal tube. After one lung ventilation, the location and length of the single incision was decided according to the purpose of the surgery. All the patients except those who underwent lobectomy for lung cancer received a 2.5 cm or 3 cm long skin incision, which was enough for the SITS technique to be applied. In case of lobectomy for lung cancer, the size of the lung specimen was needed to be taken into consideration. Thus, we made a 4 to 5 cm long incision in the mid-axillary line of the 5^th^ intercostal space (ICS). When approaching to the upper lobe such as cases of PSP, the incision was made on the 5^th^ ICS in the mid-axillary line. However, when approaching to the lower lobe, the 6^th^ or 7^th^ ICS in the anterior axillary line was selected. If the patient had a previous wound scar for VATS or closed thoracostomy, we actively utilized the site despite of surgeon’s discomfort.

**Figure 1:**
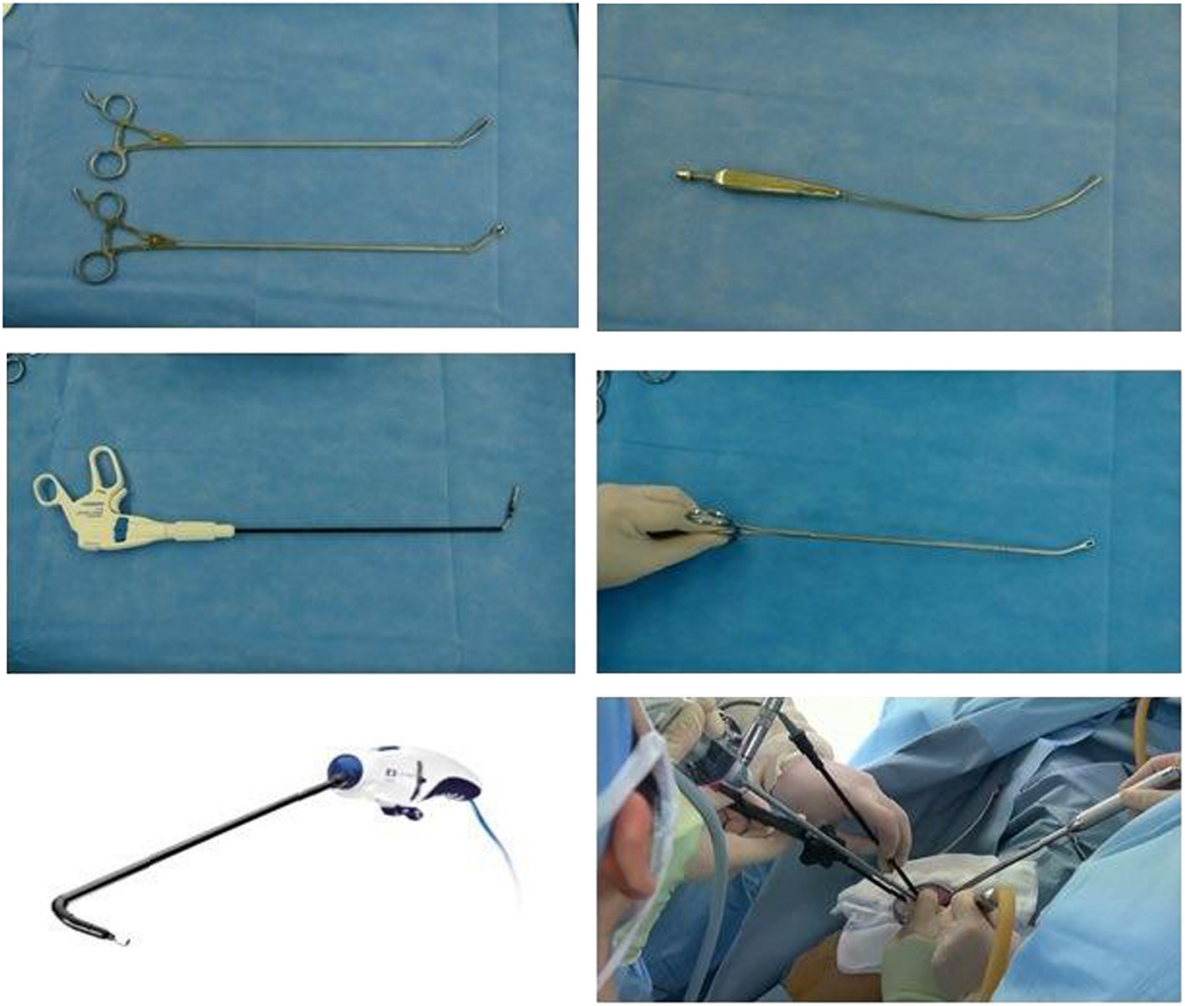
Instruments for single incision thoracoscopic surgery.

The same pain control method was used in all the patients. Bupivacaine of 0.5% with 10 to 15 cc was injected on the parietal pleura, muscle, and subcutaneous layer before skin incision. Continuous-patient-controlled analgesia was not used for pain control. We created small utility window by placing wound retractor (XS U-tractor™, YUWON MEDITECH, Wonju, Korea) to maintain air venting and block optical lens contamination by intercostal muscle bleeding. In all cases, a 5.5-mm extended length HD Autoclavable thoracoscope, 30-degree (Conmed, Utica, NY, USA) was used. We utilized the flexible or long curved thin shaft instruments in order to minimize conflicts between instruments and smoothly carry out the procedures (Figure 1). We frequently changed between hook type and spatula type electrocautery for hilar dissection as appropriate during the operation, and used LigaSure™ (Covidien, Mansfield, MA, USA) if necessary. In lobectomy cases, we divided pulmonary vein and bronchus with endo-staplers. If the pulmonary artery branch was less than 5 mm, we divided it using the locking clips, but if the pulmonary artery was larger than 5 mm, we used endo-stapler. Patients who underwent lobectomy for lung cancer underwent systematic lymph node dissection. For cases of pneumothorax, we performed mechanical pleurodesis together with wedge resection in the beginning. However, we are now routinely using fibrin glue coverage due to the side effects of mechanical pleurodesis such as postoperative pain, paresthesia, and the risk of bleeding.

Empyema decortication was performed for fibrinopurulent or organized stage after chest tube drainage treatment had failed to achieve the desired results. We aimed to drain the exudative fluid and remove the empyema sac and thickened peel to restore lung expansion. Successful cases of empyema decortication by SITS were those that had good peel removal at an appropriate time point.

If a second port is required due to extensive pleural adhesion, a 3 cm length of utility window was made to enable the finger dissection. In the situation of difficult endo-stapling, an additional port was made to allow the easy stapling movement with the size of 1.5 cm.

**Figure 2:**
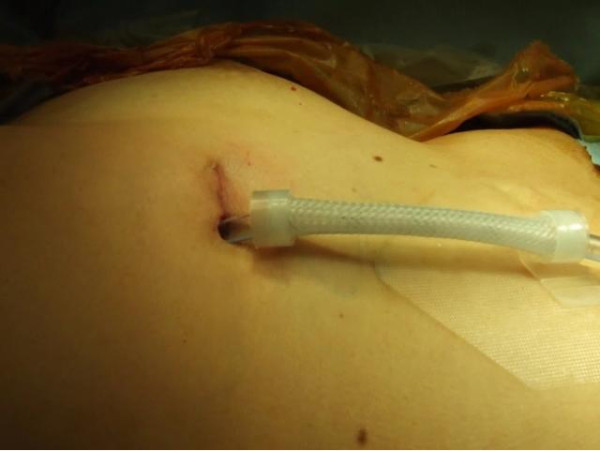
Single port right upper lobectomy with only 2.5 cm skin incision.

One chest tube (20 or 24 french chest tube) was inserted through one end of the single incision (Figure 2). Exceptionally, in the case of empyema, we inserted two chest tubes through each end of the incision.

The decision to remove the chest tube was made by the following criteria: the lungs fully expanded and if there is no pleural effusion in the chest x-ray; there is no air leakage through the chest tube; the amount of drainage is less than 150 cc per day. In the cases of pneumothorax, most patients had chest tube removal on postoperative day 1 and discharged on that day after confirming the postprocedural chest x-ray.

### Statistical analysis

Data were analyzed with SPSS 18.0 software program (Statistical Package for Social Sciences, SPSS Inc., Chicago, IL, USA). Continuous variables were presented as median value with range.

## Results

SITS was attempted in 257 patients. Seven of these patients underwent surgery twice; so a total of 264 cases of SITS were tried. A number of thoracic diseases and procedures, including PSP (n = 172), secondary spontaneous pneumothorax (SSP) (n = 22), lung biopsy (n = 29), lobectomy (n = 6), mediastinal mass excision (n = 11), empyema decortication (n = 11), pulmonary metastasectomy (n = 3), mediastinal lymph node (LN) biopsy (n = 3), pericardial window formation (n = 3), pleural biopsy with talc pleurodesis for malignant pleural effusion (n = 3), and postoperative hematoma evacuation (n = 1), were attempted with SITS. Among them, SITS was performed successfully in 237 cases (89.8%) without additional incision.

The median age was 23 years old (range: 12–86) and the median operation time was 75.5 minutes (range: 30–285). The median postoperative hospital stay was 2 days (range: 1–37), and the median follow-up duration was 5.1 months (range: 0.1-24) (Table 2). In 27 cases (10.2%) additional incisions were needed. The reasons of additional incision were as follows: extensive pleural adhesion (n = 14, 5.3%), difficulty in approaching with an endoscopic stapler (n = 11, 4.1%), bleeding (n = 1, 0.4%), intolerance of one lung ventilation (n = 1, 0.4%) (Table 3). Conversion rate of empyema was 54.5%, which was the most difficult disease entity applied to SITS. In contrast, the conversion rate of PSP was 4.7%, which means PSP was the most applicable for SITS. All lobectomies (n = 6) were performed without additional incision.

**Table 2:** Patients characteristics

**Variables**	**SITS (n = 237)**	**Conversion (n = 27)**	**Total (n = 264)**
Sex (M/F)	175/55	18/9	193/64
Age, years	22 (12–86)	57 (15–81)	23 (12–86)
Operation time, min	73 (30–260)	114 (45–285)	75.5 (30–285)
Hospital stay, days	2 (1–36)	3 (1–37)	2 (1–37)
Follow-up period, months	4.4 (0.1-24)	9.2 (1.5-20.1)	5.1 (0.1-24)

Continuous variables were presented as median value with range.

SITS, Single Incision Thoracoscopic Surgery; M, male; F, female; min, minute.

**Table 3:** Reasons for additional incision

**Reason**	**Empyema**	**ILD/SPN**	**PSP**	**SSP**	**Mediastinal tumor**	**Mediastinal LN enlargement**	**Total (n = 27)**
Pleural adhesion	5	4	3	1	1	NA	14
Location of lesion	1	1	5	3	NA	1	11
Bleeding	NA	NA	NA	NA	NA	1	1
Difficult one lung ventilation	NA	1	NA	NA	NA	NA	1

ILD, Interstitial Lung Disease; SPN, Solitary Pulmonary Nodule; PSP, Primary Spontaneous Pneumothorax; SSP, Secondary Spontaneous Pneumothorax; LN, Lymph Node; NA, Not Applicable.

Table 4 showed age, operation time, length of postoperative hospital stay, postoperative complication, follow-up period, early outcome according to each SITS procedure. Operation time and hospital stay in the patients with simple pulmonary wedge resection including pneumothorax, interstitial lung disease, or solitary pulmonary nodule were shorter than other SITS procedures. In contrast, lobectomy showed the longest operation time, and decortication patients had the longest hospital stay. Postoperative complication occurred in 7 (7/237 = 2.95%) patients. One PSP patient underwent hematoma evacuation due to postoperative bleeding from parietal pleura after pulmonary wedge resection and mechanical pleurodesis. One patient had air leakage for more than 3 days. Two patients had wound dehiscence, which was solved with skin stapler. One patient who underwent left lower lobectomy readmitted due to increased pleural effusion and was treated with 12 French mini-chest tube insertion. One PSP patient with bilateral multiple bullae had wound infection and contamination of thoracic cavity after receiving operation on both sides. This patient was originally transferred to our hospital on the 7^th^ day of chest tube insertion from the other hospital because he had prolonged air leakage with moderate subcutaneous emphysema. We believe this may have led to bacterial infection carried from the previous chest tube insertion site. SITS irrigation was performed on bilateral thoracic cavities on postoperative day (POD) 6 and discharged without any other problem on POD 8 of redo-SITS. Another PSP patient was diagnosed with wound infection on POD 19 in outpatient visit. Wound infection was treated with simple dressing for 4 days.

**Table 4:** Comparisons among major operative methods in SITS

**Variables**	**Wedge resection, PNX (n = 194)**	Wedge resection, others* (n = 32)	**Lobectomy (n = 6)**	**Decortication (n = 11)**	**Mediastinal mass excision (n = 11)**
Median age, years	19 (14–79)	61.5 (34–86)	50 (12–66)	59 (38–77)	45 (13–69)
Median Operation time, min	75.5 (34–208)	64.5 (30–165)	219 (160–260)	175 (84–285)	93 (39–175)
Median postoperative hospital stay, day	2 (1–14)	4 (1–36)	3.5 (3–6)	8 (5–26)	2 (1–3)
Postoperative complication	Air leak		Pleural		
	(n = 1)		Effusion		
	Wound dehiscence		(n = 1)		
	(n = 2)				
	Infection (n = 2)				
	Bleeding (n = 1)				

Median F/U period, month	5.1 (0.1–24)	5.35 (0.8–18.4)	2.55 (0.3–9.4)	3.6 (1.5–12.6)	6.8 (0.7–22.7)
Early outcome (recurrence)	10 (5.2%)		NED	NED	NED

*Others included interstitial lung disease, solitary pulmonary nodule, pulmonary metastases and tuberculosis.

PNX, Pneumothorax; min, minute; F/U, follow up; NED, No evidence of disease.

During the follow-up of the patients with PSP and SSP with median 5.1 months (range: 0.1-24), there were 10 pneumothorax recurrences (5.2%). Two patients underwent reoperation, four patients recovered after fibrin pleurodesis via chest tube. The remaining 4 patients had minimal sign of pneumothorax, which needed just observation. There was no recurrence in the patients with empyema and lung cancer.

## Discussion

Most of the published studies are only case reports or papers outlining how to perform SITS [5-7,9,10,12,14]. Also comparative studies with the conventional 3-port VATS were performed in the aspects of the safety and efficacy only in a small case series [5,7]. Recently, Gonzales, who has most experience with SITS, reported 97 patients undergoing uniportal VATS lobectomy [6].

This study was set up to assess whether SITS can be acceptable as the routine procedure in the thoracic surgery. A large scale of our study suggests SITS can be a safe and feasible alternative for the conventional 3-port VATS. The major obstacles of SITS were the extensive pleural adhesion and access difficulty for pulmonary wedge resection with endoscopic stapler.

We have first introduced the SITS technique in PSP patients. Since then SITS was considered as a routine procedure in all pneumothorax patients including SSP. We recommend the PSP as the first possible surgical consideration for SITS. This is because this disease has no pleural adhesion problems, and the blebs or bullae usually develop at apical portion of upper lobe alongside the coronal plane, which starts from the apicolateral portion of the upper lobe to the medial side. The distinctive feature of this disease makes wedge resection possible without hindrance. In other words, the stapler is inserted through the 5^th^ ICS in the mid-axillary line, and we can easily perform wedge resection from the lateral to the medial side around the apical portion of the upper lobe. These clinical experiences helped us broaden application of SITS not only in pulmonary wedge resection, but also in mediastinal mass excision, lung/LN/ pleural biopsy, empyema decortication, pericardial window formation, and lobectomy. Moreover, through the accumulation of these experiences, SITS is demonstrated as a safe and feasible method that can be selected as a routine procedure.

As stated previously, SITS was performed mostly in both PSP and SSP. Currently, SITS has become the most preferred method in the pneumothorax surgery. The conversion rate from SITS to 2-port VATS for PSP and SSP was 6.2% (12/194). However, such conversion mainly occurred in the initial learning period due to lesion location (n = 8) and pleural adhesions (n = 4). Currently, SITS is being carried out with very little conversions. With regards to pleural adhesiolysis, the use of SILS™ HOOK (Covidien, Mansfield, MA, USA) proved to be very useful.

Some PSP patients had two incisions despite of SITS technique because we could not utilize initial chest tube insertion site located on the 6^th^ or 7^th^ ICS, which was made in the Emergency Department. These days, residents are instructed to perform chest tube insertion properly, with consideration to the possibility of SITS later. With this, we are carefully attending to reduce the unnecessary scars endured by patients.

As seen in our results, the second biggest reason for the need of additional incision was the difficulty in accessing the lesion for endoscopic stapler. To overcome this, proper incision location is very important for SITS. We were able to reduce the conversion rate via making the small utility window based on the purpose of surgery and location of lesions. In the case of wedge resection on the upper lobe or superior segment of the lower lobe, it is better to locate the incision on the 5^th^ ICS mid-axillary line. In case of wedge resection on basal portion of the lower lobe, the 6^th^ or 7^th^ ICS in the anterior axillary line is appropriate. Although it may be different based on patients’ condition, in case of wedge resection, the incision for the port should be made 6-7 cm apart from the lesion in order to use a 60 mm length stapler. If it is difficult to obtain the stated distance, using a 45 mm length stapler may be useful.

Based on the type of disease, empyema decortication had higher conversion rate (6/11, 54.5%) because safely dissecting the extensive pleural adhesion via single port was difficult. In case of extensive and tight pleural adhesion, like empyema, 2 or 3-port VATS is better suited than SITS.

Our study included six lobectomies. As similar to previous studies, a single port lobectomy was possible on the 5th ICS in anterior to mid axillary line [6] (Figure 2). Since it was easier to access the hilar structure, we were able to quickly get accustomed. In the 3 cases of lung cancer, 4-5 cm of skin incision was necessary to extract lobar specimen without injury. In other 3 patients who underwent lobectomy for benign lung disease, including bronchiectasis, aspergilloma, and congenital lobar emphysema, the skin incision was minimized to only 2.5 cm. On our first case of a 2.5 cm lobectomy, we cut the lobe into 3 pieces in a specimen bag with scissors. However, on our second and third cases, a 2.5 cm skin incision was successfully used to extract the lobar specimen without significant injury. Thereafter, we realized that in cases with benign diseases without a mass, even with a 2.5 cm skin incision, it is possible to extract the lobe without the damages to the lung.

Some authors defined that length of single port was less than 3.5 cm [16]. But there is no consensus on the length of single incision to be considered as SITS. The factors that determine the length of skin incision are the size of the specimen and whether the stapler is used or not. From our experience, 2.5 cm was the minimum incision length when stapling was needed. However, 2 cm was enough when stapling was not needed.

For successful outcome of SITS, it is essential to properly set the equipment for SITS. We used a wound retractor to maintain the utility window to prevent optical lens blood contamination from the chest wall muscle and to eliminate smoke from electrocautery. In regard of preventing instrument conflict, long and narrow shaft, and curved or flexible instruments were seemed to be helpful. In the future, if curved and smaller stapler is developed, the applicability of SITS will be expanded.

The advantages of SITS over the 3-port VATS are less pain, less paresthesia, and higher satisfaction rate for wound scar [5-12,14,16]. Despite of these advantages, there are still very limited numbers of surgeons who perform SITS. However, many young surgeons express great interest in learning the application of SITS in various diseases. Although we only had 6 lobectomy cases, we have the large number of experiences with SITS for various diseases. We believe that this study will help those who seek to start incorporating SITS as a routine approach.

Cosmesis is an important aspect of SITS. In order to reduce wound problem, we minimize electrocauterization on chest wall muscles to make a single port, which helps the spontaneous approximation of the muscle layer in the site of chest tube after removal of the chest tube. We also use 20 or 24 Fr. chest tube and do our best to shorten the chest tube indwelling time. Skin bond is an effective prevention for wound dehiscence. These efforts have helped us overcome the wound problems.

There are a few limitations to our study. First, the follow-up period is relatively short because it has been only 24 months since SITS was performed at our hospital. Thus, long-term follow up data for recurrences of pneumothorax or oncologic results in lung cancer patients are not enough. Second, a majority of the patients (165/257, 64.2%) were diagnosed with PSP. Hence, further cases should be performed around other disease entities. This is because the surgeries were carried out by a single operator (HCY), who is mainly in charge of benign thoracic diseases. However, two hundred fifty seven patients underwent SITS during 24 months. This was possible because the operator considered SITS as the first routine procedure for the thoracic patients. Third, this is not a comparative study with 3-port VATS. Therefore, there is no evidence to demonstrate SITS as a superior method compared to the conventional VATS. However, in our experience, the SITS sufficiently satisfied what the conventional 3-port VATS has aimed. In addition, it involves only one intercostal space with minimal skin incision, which means SITS is the most minimally invasive surgery known until today. To prove the advantages of SITS more clearly compared to 3-port VATS, a multicenter, large-scale randomized controlled study should be performed in the near future.

## Conclusions

In conclusions, for successful SITS, well-equipment, location of port, experiences in simple thoracic procedures and harmony with surgical assistants are necessary. SITS is considered as a safe, feasible and reproducible procedure in variety of thoracic diseases. However, in case of extensive pleural adhesion, as in empyema, or location of lesion with difficult accessibility for stapling, additional incisions may be safer for patients.

## Abbreviations

SITS: Single incision thoracoscopic surgery; VATS: Video-assisted thoracic surgery; PSP: Primary spontaneous pneumothorax; SSP: Secondary spontaneous pneumothorax; ICS: Intercostal space; TB: Tuberculosis; LN: Lymph node; CT: Chemotherapy; ILD: Intersitial lung disease; SPN: Solitary pulmonary nodule.

## Competing interests

The authors declare that they have no competing interests.

## Authors’ contributions

IS participated in clinical practice, contributed to collection and analysis of data, drafting the manuscript, and revising it. SY carried out data collection. WC participated in clinical practice. SC, KK and SJ helped in design of study and drafting the manuscript. HCY carried out patient recruitment and clinical practice, contributed to conception, design, drafting the manuscript, and revising it. All authors read and approved the final manuscript.
